# Examining the Influence of Historical Redlining on Firearm Injuries in Current Day Baltimore, Maryland

**DOI:** 10.21203/rs.3.rs-4534823/v1

**Published:** 2024-06-25

**Authors:** Marianne So, Mahmoud G El Baassiri, Matthew D Price, James P Byrne, Elliott R Haut, Isam W Nasr

**Affiliations:** Johns Hopkins Children’s Center Division of General Pediatric Surgery; Johns Hopkins Children’s Center Division of General Pediatric Surgery; Johns Hopkins Department of Surgery: Johns Hopkins Medicine Department of Surgery; Johns Hopkins Department of Surgery: Johns Hopkins Medicine Department of Surgery; Johns Hopkins Department of Surgery: Johns Hopkins Medicine Department of Surgery; Johns Hopkins Children’s Center Division of General Pediatric Surgery

## Abstract

Firearm injuries are a common and major public health problem in Baltimore, Maryland. The city is also one of the first U.S. cities in which the 1930s discriminatory practice of redlining first emerged. This study examines the association between current day firearm injuries and residence in these historically redlined areas at a neighborhood level using zip codes. Firearm injury outcomes in patients who presented to a hospital in Maryland from 2015 to 2020 were measured from the Health Services Cost Review Commission (HSCRC) in conjunction with both geospatial data from Richmond’s Digital Scholarship Lab’s Mapping Inequality project and population data from the U.S. Census. A redlining score was calculated to represent the extent of redlining in each zip code. Negative binomial regression models were utilized to measure the association between neighborhood zip codes and rate of firearm injuries. Our adjusted regression model shows that for every one-unit increase of the Home Owners’ Loan Corporation (HOLC) redlining score, there is a 2.24-fold increase in the rate of firearm injuries (RR 2.24; 95% CI: 0.31, 1.31, p < 0.001). These findings suggest a strongassociation between historically redlined areas and population risk of firearm injury today. Further research is needed to investigate the underlying mechanisms that may contribute to this relationship, such as access to firearms or social and economic factors. Overall, our study highlights the potential impact of historical redlining policies on contemporary health outcomes in Baltimore.

## Introduction

Firearm injuries represent a pressing public health challenge in the United States, with recent increases in both fatalities and nonfatal incidents (1). These injuries disproportionately affect marginalized communities, particularly people of color and young males (2). Such disparities underscore broader systemic issues of inequality and social injustice. To understand the roots of these disparities, it is crucial to examine historical factors such as redlining, which have had enduring impacts on contemporary health outcomes (3).

Originating in the 1930s, redlining was a discriminatory practice that systematically denied financial services, including mortgages, to neighborhoods based on racial demographics (4). This practice reenforced racial segregation and perpetuated economic disparities, as predominantly Black neighborhoods were often designated as high-risk areas for mortgage and lending purposes (5). Baltimore, with its documented history of discriminatory housing policies, serves as an example of the lasting effects of redlining. Despite legal reforms, the legacy of redlining continues to shape the city’s landscape, contributing to persistent socioeconomic challenges and health disparities (6).

Baltimore, characterized by its predominantly Black population and high poverty rates, faces a complex array of public health challenges, including firearm-related violence (7). Baltimore has the second highest firearm-related mortality rate in the United States, with an alarming rate of firearm homicides and nonfatal shootings (6). The city’s history of discriminatory practices has led to significant health inequities, firearm violence and socioeconomic difficulties (8). In one study assessing the effect of redlining on firearm injuries, Baltimore was found to have the highest association (9).

Our study seeks to identify the association between historical redlining and firearm injuries in Baltimore. By examining neighborhood-level data on redlining grades alongside firearm injury rates, we aim to identify the mechanisms through which historical housing policies continue to influence health outcomes today. Through this analysis, we aim to inform targeted interventions and policy initiatives aimed at addressing the root cause of firearm injuries in marginalized communities.

## Methods

### Study Design and Datasets:

The Maryland Health Services Cost Review Commission (HSCRC) is an independent state agency that has a data repository containing administrative hospital data for the state of Maryland. The HSCRC includes deidentified patient-level data for all inpatient discharges and emergency department visits.

The Home Owners’ Loan Corporation (HOLC) was commissioned in the 1930s as part of the federal government’s efforts to provide housing and mortgage relief. Over 200 American cities had “residential security maps” drawn, detailing the level of risk of lending for mortgages in various neighborhoods within each city. These cities include places such as Chicago, Detroit, Philadelphia, San Francisco, New York, and Baltimore (1). Areas on the map were given a possibility of four different grades of A, B, C, and D, and were respectively designated by four colors, green, blue, yellow and red. The level of lending risk are as follows: green-colored areas (A) were “Best”, blue-colored areas (B) were “Still Desirable”, yellow-colored areas (C) were “Definitely Declining”, and red-colored areas (D) were as “Hazardous”.

Baltimore’s digitized HOLC map were obtained from the Mapping Inequality project by Richmond’s Digital Scholarship Lab. The map represents the 1930’s HOLC grades that demonstrate the degree of redlining in Baltimore and were read as a standard geospatial data interchange format that encodes geographic data structures.

### Study Population:

We used the HSCRC database to identify all firearm injuries who presented to a hospital in Baltimore, Maryland from October 1, 2015 to December 30, 2020 using ICD-10 diagnosis codes for firearm injuries (W32, W33, W34, W72, X73, X74, X93, X94, X95, Y22, Y23, Y23, and Y35). This excludes firearm injuries that occurred but were not seen in a Maryland hospital setting (including deaths and/or minor injuries). As most of the redlined areas are in Baltimore, only patients with zip codes within the city limits were included in the study. Records which had missing zip codes, contained zip codes outside of Baltimore, and were not Maryland residents were excluded. A total of 36 Baltimore zip codes were used for our analysis.

### Outcome measure:

The primary outcome is to examine whether living in Baltimore’s redlined areas is associated with higher rates of firearm injury. Our dependent variable is the count of healthcare encounters for firearm injuries that occur in each contemporary zip code of Baltimore and will be offset by the overall number of people that live in that zip code as provided by the U.S. Census. Using a negative binomial regression model, we examined the rate of healthcare encounters for firearm injuries in relation to historical HOLC grades. Chi square analyses were performed on demographic variables describing our study population, with a significance level p < 0.05. Our final adjusted regression model was based on running multiple univariable and multivariable models in a stepwise manner and evaluating each model’s AIC scores. All statistical analyses were performed using R statistical software (Version 4.2).

### Redlining Identification and Geospatial Analysis:

Baltimore’s digitized HOLC maps were transformed into data frames using tidy and broom packages in R. After the creation of these data frames, they were plotted as colored polygon shapefiles based on geographic coordinates. Since this HOLC grade data is only available on a census tract level and since census tracts are more granular geographic entities than zip codes, we imputed the HOLC grade data to correspond with current Baltimore zip codes which allowed for an alignment with the zip code residence data provided by HSCRC. This spatial smoothing of census tract data to zip code level data allows us to examine commensurate HOLC associations by zip code.

The polygons represent the four graded HOLC designations and zip code boundaries. This HOLC map was then overlayed over another map containing Baltimore zip codes corresponding to zip code tabulation areas (ZCTAs) from the U.S. Census and the Mapping Inequality project. One issue commonly faced in similar studies is the difference in geographic units of analysis, as census tracts and zip codes do not have perfectly align with one another. However, given that ZCTAs are generalized areal representations of U.S. Postal Service zip codes, have very little differences from each other, and have been used as statistical entities by the U.S. Census, we chose to link ZCTAs and zip code data together.

To determine the boundaries and get a percentage value of the overlaps from the HOLC and zip code/ZCTA data, spatial analysis packages were used to estimate the overlap of the two polygon maps. HOLC IDs were linked to zip codes by area of land in meters squared. We then converted zip code groups to percentages using a columnwise sum and divided by the land areas to compute the columnwise average of HOLC coverage. HOLC letter grades were assigned quantitative units to allow for a measurement of redlining on a linear scale. The grade designations are as follows: A = 1, B = 2, C = 3, and D = 4. Areas with the higher redlining scores indicate higher HOLC grades of redlining.

### Statistical Analysis:

Chi-square tests were used to evaluate differences in demographic characteristics. Univariable comparisons between redlined and non-redlined zip codes were conducted, followed by a negative binomial regression model to assess the association between HOLC grades and firearm injury. Final model selection was based on AIC scores. All analysis were performed using R statistical software (Version 4.2)

Median income, education, and ethnicity were excluded from the adjusted negative binomial regression model due to its high collinearity with our outcome variable of firearm injury. The decision to exclude these variables was made due to multicollinearity issues. Excluding these variables further ensured the validity of this study’s model results. These exclusions may limit our interpretations of our findings as income, education, and ethnicity have been identified as important predictors of firearm injuries. Lastly our analysis is cross-sectional, meaning that we cannot establish causality and can only infer that an association exists between redlining and firearm injuries.

Several negative binomial regression models were examined on a univariate and multivariate level. Population size was controlled in all models and included as an offset parameter for our models. Based on the study population demographics and the current body of literature on redlining in Baltimore, our covariate of interest was median age (6, 8, 10–12). Once univariate models were performed by zip code with their corresponding redlining scores, the coefficients, intercepts, and AIC score were compared with one another.

## Results

**Figure 1:**
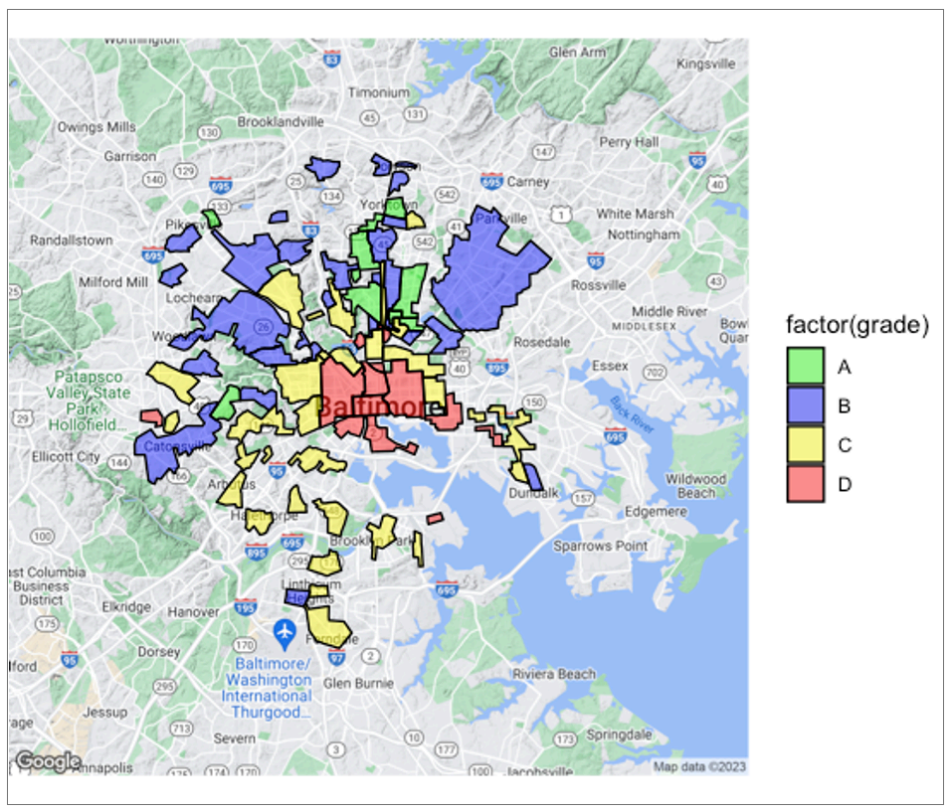
Present-day map of Baltimore with historically redlined areas by HOLC grade.

A total of 1055 patients and 36 zip codes were analyzed in our study population. Figure 1 depicts a present-day map of Baltimore with historical redlined areas. The colors and grades correspond to the areas of redlining as originally depicted by the HOLC, with green being the lowest grade (A) and red being the highest grade (D).

The majority of those who suffered a firearm injury were male (91.5%), which is 11 times higher than firearm injuries observed in females. 47.5% of firearm injuries occurred in individuals between 15–34 years of age. 94.8% identified as not ethnically Spanish/Hispanic (Table 1). The majority identified as African American (87.1%), followed by 8.6% who identified as White. Individuals who identified as mixed race, non-White, or non-African American (4.3%) were categorized in the “Other” category. When examining insurance status, 87.2% noted that they did have insurance. Most of those who did have insurance were insured under Medicaid or Medicare.

**Figure 2:**
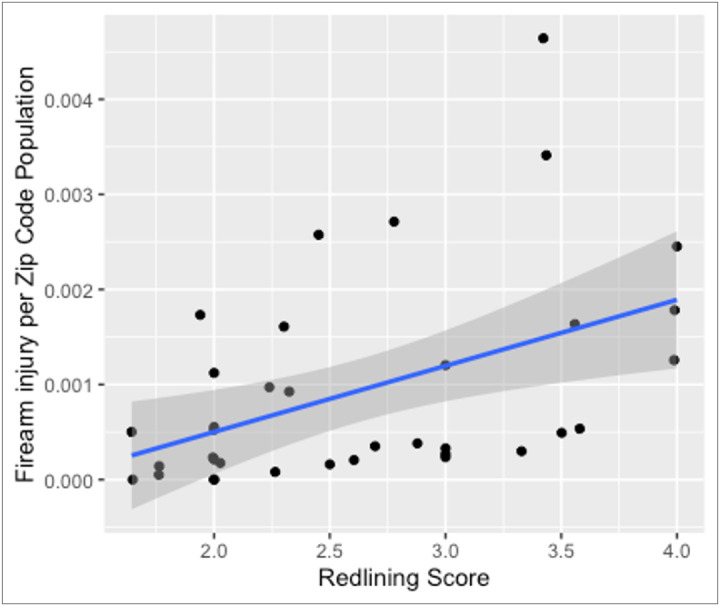
Firearm injury rate scatterplot of zip code by redlining score.

HOLC grades were quantified with A = 1, B = 2, C = 3, and D = 4. The mean firearm count per zip code was 29.31 with a median of 17.50. Redlining scores ranged from 1.64 to 4 (Figure 2). There was a strong association between increased redlining score and firearm injuries per population (r=0.49, p=0.0003). Firearm injuries by zip code population increased as the redlining score increased.

Most firearm injuries (74.6%) were assaults by handgun discharge, rifle, shotgun, a larger firearm, or other unspecified firearm or gun where as 21.5% were a result of accidental injuries and 3.9% of undetermined intent (Table 2).

Descriptive statistics of the rate of firearm injury in each zip code can be found in Table 3, along with the HOLC grade and redlining score for each zip code. Zip code 21217 was found to have the highest number of firearm injuries (112). Zip codes 21201, 21202 and 21231 had the highest redlining score=4 while zip code 21201 had the lowest redlining score=1.6.

Unadjusted and adjusted negative binomial regression model analyses of firearm injury count were performed with redlining score and median age as our covariates of interest (Table 4). In our unadjusted univariate models, we found that for every one unit increase in redlining score, there is a statistically significant 2.054 fold increase in the rate of firearm injuries (p=0.001). However, median age was found to be statistically insignificant (p=0.296). In our multivariate adjusted models, our results indicate a statistically significant strong relationship between HOLC scores and firearm injury rates (p = 0.000823). For every one unit increase of the HOLC score, there is a 2.247-fold increase in the rate of firearm injuries. This finding show that zip code areas with higher HOLC scores have higher rates of firearm injury.

## Discussion

In this study, we examined the relationship between redlining and firearm injuries in Baltimore from 2015 to 2020. The results showed a strong association between HOLC scores and firearm injuries suggesting that historically redlined areas are disproportionately affected by firearm injuries even today, 100 years after redlining began. These findings are consistent with previous research that has shown a link between redlining and various health outcomes (6, 11, 13–15). In one previous study, Huang et al. found that HOLC redlining was associated with a decrease of life expectancy for those living in these regions (6). This mortality could be due to crime as well as poorer health outcomes that are a direct result of redlining. In another study examining redlining in Baltimore, Uzzi et al. found an increase in firearm injuries between 2015 and 2019, where the interaction between redlining and racialized economic segregation explained over one-third of non-fatal shootings (approximately 650 shootings) in sustained disadvantaged areas (8).

The demographic characteristics of our study population also shed light on some of the disparities underlying firearm injuries in Baltimore. The majority of those who suffered a firearm injury were male, young adults between 15–34 years of age, and Black (Table 1) with the most common type of firearm injury caused by handgun discharge (69.9%) (Table 2). These findings align with national statistics showing firearm injuries disproportionately affect young males and people of color (16). Furthermore, our results indicate that the highest number of firearm injuries was concentrated in zip code 21217 (HOLC grade D), where 112 firearm injuries were recorded (Table 3). Notably, this zip code encompasses the Sandtown-Winchester neighborhood, where Freddie Gray lived before his tragic death. Freddie Gray, a 25 year old black man, suffered a spinal cord injury and was killed in police custody in 2015. This incident of police mistreatment of black men has been also linked to the city’s history of redlining, further emphasizing the connection to the clustering of firearms in these areas (8, 17). In addition, our study found that a large proportion of firearm injuries were due to assaults with handguns and other firearms (Table 2), highlighting the need for violence prevention measures that address the root causes of firearm injuries.

Without applying a historical and contemporary lens, many may blame current disparities on the very people residing in communities with high crime and violence, when in fact, other contributing factors outside of their control are at play (18). This belief can lead to a reticence in areas in need of revitalization and investment, keeping these communities in a cycle of poverty. Continued disinvestment has also increased the establishment of tobacco and alcohol outlets in impoverished neighborhoods that lack things that add value and safety to a community, such as quality food, fair housing, and green spaces (11). For example, studies have shown that the clustering of alcohol outlets is more congregated in areas where residents have a lower socioeconomic status and are often located near vacant or abandoned housing (12, 14).

Our findings suggest that for every one unit increase of the HOLC score, the rate of firearm injuries increases by a multiplicative factor of 2.054 (Table 4). Additionally we found that a higher number of firearm injuries occurred in areas with HOLC grades of C and D. These results suggest that the redlining practices of the past continue to have long-lasting effects on health and wellbeing of those who reside in these areas today. Redlining continues as part of a broader national conversation about the impact of racially motivated discriminatory policies and systems that have led to systematic racial discrimination in housing. These events have had long-lasting impacts on intergenerational wealth, access to fair housing, and overall quality of life in Black and marginalized communities (19). These communities continue to face systemic barriers to homeownership and access to credit (3).

Our study has several limitations that should be acknowledged. Firstly, our data is based on hospital records and may not capture all firearm injuries that occurred in the population. Because the HOLC geospatial data was only available by census tracts, the data had to be transformed into various forms before it was workable with our zip code level data as provided by the HSCRC. Additionally, we also supplemented this study by utilizing U.S. Census population data. To keep the data as similar as possible to its original geographic unit, we decided to utilize ZCTA data from the U.S. Census. Based on existing literature that demonstrates a high degree of similarity between zip code and ZCTA data and that ZCTAs are designed with the purpose of replicating currently existing zip code areas, we assumed ZCTAs, and zip codes are one in the same for the purposes of data analysis.

Ecological fallacies should be considered as they rely on models based on historical data in conjunction with present data. Since we studied firearm injuries on aggregate-level data, information about individual-level behavior may be lost in that aggregation and not represented in our data. Additionally, firearm injury rates are influenced by several factors that were not measured in this study, such as criminal activity and access to firearms. These rates can be influenced by seasonality or global events. For example, studies have shown that firearm injury rates are higher during the summer, when temperatures are warmer (20). Additionally, firearm injury rates were also observed to have increased during the COVID-19 pandemic, when people were forced to stay at home and self-quarantine or isolate themselves (21). This was likely due to increased firearm purchases, reduced law enforcement presence, economic instability, increased incidences of domestic violence due to closer living proximities and increased interpersonal conflicts (22).

Therefore, it is suggested that these study findings only be used as a frame of reference and should consider current social and political contexts of Baltimore. Additionally, geoanalysis with HOLC data in comparison to U.S. Census tracts, zip codes, and ZCTAs do not perfectly overlap with the HOLC maps—as a result, this required geospatial and statistical corrections. The corrections were done based on other ecological and geospatial studies examining redlining in Baltimore, as well as advisement from epidemiologists and biostatisticians at Johns Hopkins Bloomberg School of Public Health (8, 9). This may make reproducing these types of studies more challenging.

## Conclusion

In conclusion, our study found that there was a strong association between firearm injuries in those who resided within historically redlined areas in Baltimore. This further adds to the growing body of evidence that discriminatory practices, as exemplified with redlining, contribute to firearm injuries. Addressing these disparities and inequities will require comprehensive and long-term solutions that address the root causes of structural racism. Further research is needed to investigate the underlying mechanisms that may contribute to this relationship, such as access to firearms or social and economic factors. Overall, our study highlights the potential impact of historical redlining on contemporary health outcomes.

## Figures and Tables

**Table 1:** Individual demographic characteristics of Baltimore City residents who live in redlined areas and suffered a firearm injury.

	Count
**Total population**	1055
**Gender**	
Male	965 (91.5%)
Female	90 (8.5%)
**Age**	
2–14	13 (1.2%)
15–24	334 (31.6%)
25–34	403 (38.2%)
35–49	203 (19.24%)
50–64	91 (8.63%)
65+	11 (1%)
**Ethnicity**	
Spanish/Hispanic	20 (1.9%)
Not Spanish/Hispanic	1000 (94.8%)
Other	35 (3.3%)
**Race**	
White	91 (8.6%)
Black	919 (87.1%)
Other	45 (4.3%)
**Insurance status**	
Insured	920 (87.2%)
Uninsured	135 (12.8%)

**Table 2:** Summary of types of firearm injuries in ICD-10-CM code.

ICD-10 CM code	Primary diagnosis	Count (n=1055)	Percent
**W32**	Accidental handgun discharge and malfunction	168	15.9%
**W33**	Accidental rifle, shotgun and larger firearm discharge and malfunction	56	5.3%
**W34**	Accidental discharge and malfunction from other and unspecified firearms and guns	3	0.3%
**W72**	Intentional self-harm by handgun	0	0.0%
**X73**	Intentional self-harm by rifle, shotgun and larger firearm discharge	0	0.0%
**X74**	Intentional self-harm by other unspecified firearm and gun discharge	0	0.0%
**X93**	Assault by handgun discharge	737	69.9%
**X94**	Assault by rifle, shotgun, and larger firearm discharge	49	4.6%
**X95**	Assault by other and unspecified firearm and gun discharge	1	0.1%
**Y22**	Handgun discharge, undetermined intent	39	3.7%
**Y23**	Rifle, shotgun and larger firearm discharge, undetermined intent	1	0.1%
**Y24**	Other and unspecified firearm discharge, undetermined intent	1	0.1%
**Y35**	Legal intervention	0	0

**Table 3:** Descriptive statistics of firearm injuries by zip code corresponding HOLC grade and redlining score.

Baltimore Cityzip code	Number of firearm injuries	Zip code population	Percent of firearm injury by population	HOLC A grade	HOLC B grade	HOLC C grade	HOLC D grade	Redlining score
**21061**	13	55020	0.0%	0.0%	0.0%	100.0%	0.0%	3.0
**21090**	2	9715	0.0%	0.0%	39.6%	60.4%	0.0%	2.6
**21201**	43	17534	0.2%	0.0%	0.0%	0.0%	100.0%	4.0
**21202**	39	21880	0.2%	0.0%	0.0%	1.0%	99.0%	4.0
**21204**	5	21518	0.0%	0.5%	99.5%	0.0%	0.0%	2.0
**21205**	26	15900	0.2%	0.0%	0.0%	44.1%	55.9%	3.6
**21206**	58	51670	0.1%	0.0%	100.0%	0.0%	0.0%	2.0
**21207**	49	50440	0.1%	0.0%	76.1%	23.9%	0.0%	2.2
**21208**	5	35273	0.0%	23.7%	76.3%	0.0%	0.0%	1.8
**21209**	5	28621	0.0%	0.0%	97.3%	2.7%	0.0%	2.0
**21210**	0	16076	0.0%	41.7%	51.7%	6.6%	0.0%	1.6
**21211**	6	15735	0.0%	4.4%	14.4%	70.0%	11.2%	2.9
**21212**	17	33865	0.1%	40.4%	54.8%	4.8%	0.0%	1.6
**21213**	83	30596	0.3%	0.1%	40.5%	40.9%	18.5%	2.8
**21214**	12	21666	0.1%	0.0%	0.0%	100.0%	0.0%	2.0
**21215**	51	55081	0.1%	0.0%	67.5%	32.5%	0.0%	2.3
**21216**	74	28733	0.3%	0.0%	54.8%	45.2%	0.0%	2.5
**21217**	112	32830	0.3%	0.0%	6.2%	44.1%	49.7%	3.4
**21218**	80	46172	0.2%	46.3%	16.9%	33.4%	3.4%	1.9
**21222**	20	56946	0.0%	0.0%	30.4%	69.6%	0.0%	2.7
**21223**	102	21974	0.5%	0.0%	0.0%	57.8%	42.2%	3.4
**21224**	24	48770	0.0%	0.0%	0.0%	49.8%	50.2%	3.5
**21225**	40	33227	0.1%	0.0%	0.0%	100.0%	0.0%	3.0
**21226**	2	6699	0.0%	0.0%	0.0%	67.2%	32.8%	3.3
**21227**	11	33396	0.0%	0.0%	0.0%	100.0%	0.0%	3.0
**21228**	4	49438	0.0%	0.0%	82.6%	8.4%	9.0%	2.3
**21229**	74	45983	0.2%	20.5%	28.9%	50.7%	0.0%	2.3
**21230**	18	33592	0.1%	0.0%	0.0%	42.0%	58.0%	3.6
**21231**	20	15906	0.1%	0.0%	0.0%	1.3%	98.7%	4.0
**21234**	36	68678	0.1%	0.0%	100.0%	0.0%	0.0%	2.0
**21236**	8	37803	0.0%	0.0%	100.0%	0.0%	0.0%	2.0
**21237**	5	31105	0.0%	0.0%	50.0%	50.0%	0.0%	2.5
**21244**	10	36897	0.0%	0.0%	0.0%	100.0%	0.0%	3.0
**21251**	0	2040	0.0%	0.0%	100.0%	0.0%	0.0%	2.0
**21252**	1	19711	0.0%	0.0%	100.0%	0.0%	0.0%	2.0
**21286**	0	1652	0.0%	23.9%	76.1%	0.0%	0.0%	1.8

**Table 4:** Results of adjusted and unadjusted negative binomial regression models of firearm injury count.

Unadjusted analysis				Adjusted analysis			
	IRR	95% CI	p-value		IRR	95% CI	p-value
**Redlining score**	2.054	(1.309, 3.236)	<0.001	**Redlining score**	2.247	(0.314, 1.305)	<0.001
**Median age**	1.039	(0.965, 1.117)	0.296	**Median age**	1.084	(−0.002, 0.164)	0.05128
